# Neuromyelitis Optica spectrum disorder complicated with pure red cell aplasia: a case report

**DOI:** 10.1186/s12883-024-03749-2

**Published:** 2024-07-11

**Authors:** Wanqing Jiang, Jue Wang, Yu Feng, Qian Liu, Mingjun Liu, Huiying Sun, Kun Zhang, Qingyu Ji, Peifei Jia, Xuewen Liu

**Affiliations:** 1 Baotou Medical College, Baotou, 014030 China; 2 Department of Hematology, The Second Affiliated Hospital of Baotou Medical College, Inner Mongolia University of Science and Technology, Baotou, 014030 China; 3 Department of Radiology, The Second Affiliated Hospital of Baotou Medical College, Inner Mongolia University of Science and Technology, Baotou, 014030 China; 4 Department of Neurology, The Second Affiliated Hospital of Baotou Medical College, Inner Mongolia University of Science and Technology, Baotou, 014030 China

**Keywords:** Neuromyelitis Optica spectrum disorders, Aquaporin 4, Red-cell aplasia, Autoimmune diseases, Case report

## Abstract

**Background:**

Pure red cell aplasia (PRCA) in neuromyelitis optica spectrum disorder (NMOSD) has not been reported before. This study presents a patient with NMOSD who developed PRCA.

**Case presentation:**

A 54-year-old female was admitted in January 2023 for dysuria and progressive numbness and weakness of lower limbs. She had difficulty standing and walking in a straight line. Both lower limbs were positive for the Babinski and Chaddock signs. MRI showed abnormal signals in the spinal cord. Aquaporin-4-IgG (AQP-4-IgG) was positive (1:320), and NMOSD was confirmed. Intravenous immunoglobulin and methylprednisolone were given, and the symptoms improved. She received maintenance treatment with methylprednisolone tablets, and the dosage was gradually reduced. She was readmitted for fatigue, palpitations, and shortness of breath in May 2023. Bone marrow aspiration and biopsy showed elevated erythroid precursors and erythroid hypoplasia, with normal megakaryocytes and myeloid precursors. Chest CT showed no mediastinal lymph node enlargement or thymoma. PRCA secondary to NMOSD was diagnosed. Recombinant human erythropoietin was given. Her condition improved after 1.5 months, as indicated by blood cell count and imaging.

**Conclusions:**

This case suggests that PRCA can be secondary to NMOSD. A comprehensive immune function and bone marrow evaluation might be necessary if abnormal blood cells are found while managing NMOSD.

## Background

Pure red cell aplasia (PRCA) is characterized by anemia, reticulocytopenia, and significant reduction or absence of erythroid precursor cells in bone marrow, with usually normal white blood cells and platelets [1]. The causes of secondary PRCA are complex and include autoimmune diseases (considered the main cause of PRCA), thymoma, leukemia, lymphoproliferative diseases, ABO-incompatible stem cell transplantation, solid tumors, viral infections, bacterial infections, drugs, and pregnancy [1]. Neuromyelitis optica spectrum disorder (NMOSD) is a rare autoimmune disease involving the inflammatory demyelination of the nervous system. Approximately 75% of patients with NMOSD have antibodies against aquaporin (AQP)-4, a water channel expressed on astrocytes [2]. These diseases may share a common immune-related pathogenesis [3]. NMOSD can be accompanied by hematological diseases, such as iron-deficiency anemia, autoimmune hemolytic anemia, and immune thrombocytopenia [4], but cases of NMOSD combined with these disorders are rare. In addition, there are no reported cases of NMOSD combined with PRCA. Therefore, this study reported a patient diagnosed with NMOSD who later developed PRCA.

## Case presentation

**Fig. 1:**
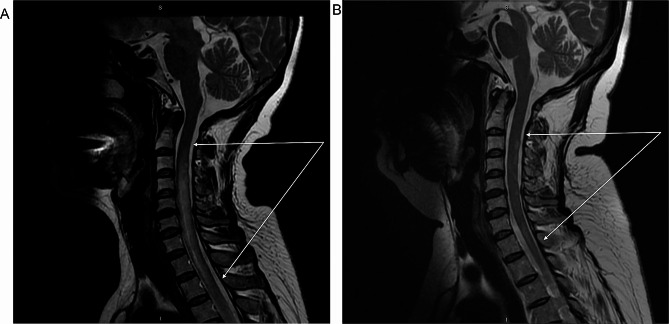
Magnetic resonance imaging (MRI) of the spinal cord. (**A**) MRI was performed when the patient was admitted to the hospital. It showed abnormal signals in the spinal cord at the C2 to T4 vertebral segments (arrows). (**B**) MRI reexamination after 4.5 months of treatments showing that the area of abnormal signals in the spinal cord has decreased (arrows)

**Fig. 2:**
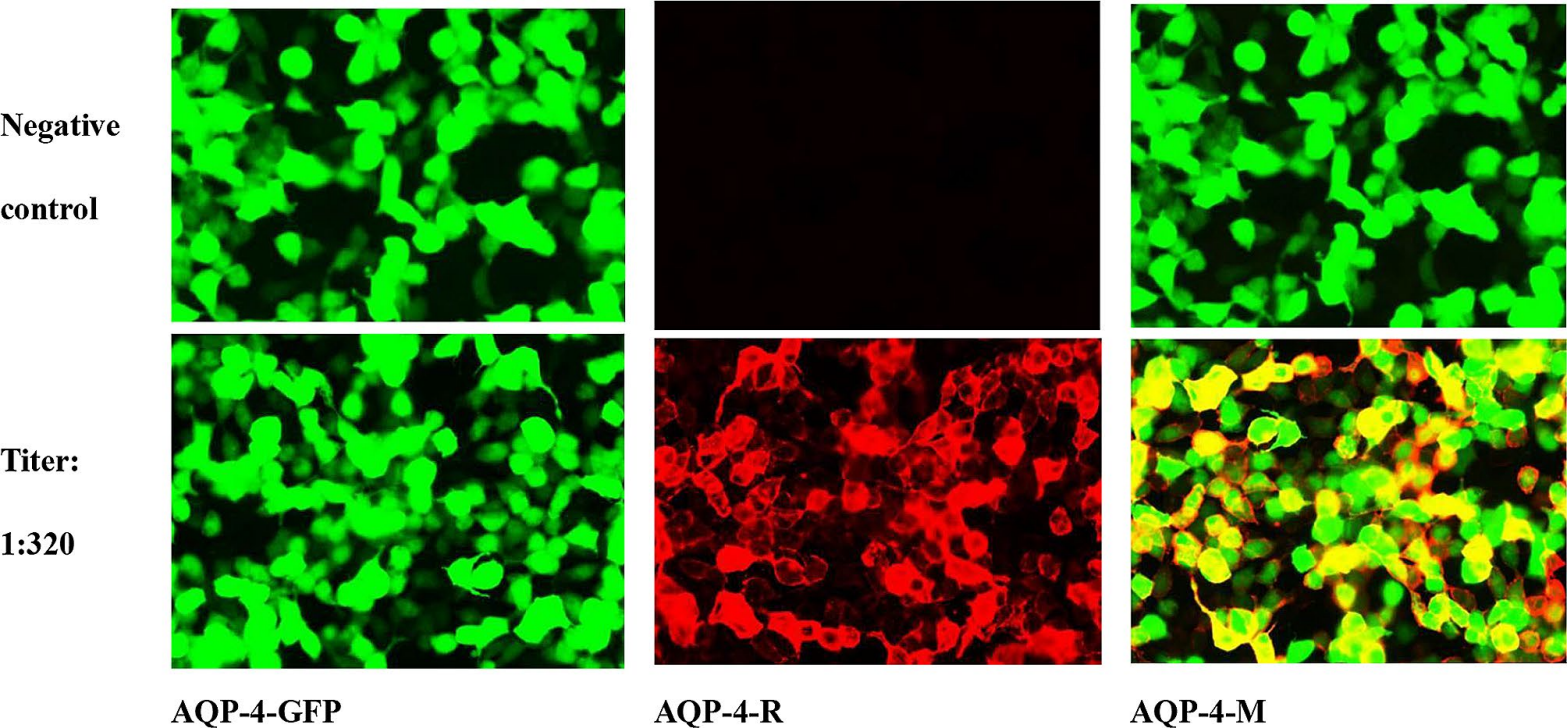
Antibodies against AQP-4 were detected in the serum using a cell-based assay (titer: 1:320). Observation with a fluorescence microscope, first observing the transfection of cells using a green light channel. If the plasmid transfection is successful, green fluorescence can be observed in the cells (AQP-4-GFP). When using a red-light channel for observation, if the membrane of the successfully transfected cells showed obvious red fluorescence (AQP-4-R), it was considered a positive sample for the antibody. Confirmation was performed by overlapping the green and red-light channels (AQP-4-M)

A 54-year-old female was admitted in January 2023 for lower limb numbness and weakness for 6 weeks, progressing to inability to walk and dysuria for 3 days before admission. She had difficulty standing when both eyes were closed and could not walk in a straight line. Skin sensation was decreased below the navel. The lower limbs showed decreased muscle strength (grade 4, MRC) and exhibited the Babinski and Chaddock signs. Brain and spinal magnetic resonance imaging (MRI) revealed Fazekas grade I leukoencephalopathy. Diffusion-weighted imaging (DWI) showed dotted hyperintense lesions on the right side of the lateral ventricles and the left side of the oval center. DWI also showed extensive longitudinal transverse myelitis in the spinal cord at the C2-T10 vertebral levels (Fig. 1A). The patient underwent a lumbar puncture. The cerebrospinal fluid (CSF) was clear and transparent, with elevated proteins, red cells, and white blood cells. CSF-IgA, CSF-IgG, and CSF-IgM were high. Plasma lactate dehydrogenase (LDH) levels were high. The AQP-4-IgG test was positive (Table 1; Fig. 2). Serum immunological examination revealed elevated antithyroglobulin antibody (TRAb), thyroperoxidase antibody (TPOAb), and anti-SSA-52. Anti-mitochondrial antibody-M2 (AMA-M2) and ANA were detected (Table 1). The patient underwent tumor screening, but no lesions were found on chest and abdominal CT scans; there were no enlarged thymus or lymph nodes. The tumor markers CEA, CA99, CA125, CA724, and AFP were normal. The diagnosis of NMOSD was confirmed. The patient was treated with intravenous immunoglobulin (IVIG, 20 g/d) and methylprednisolone (1.0 g/d). The neurological symptoms gradually improved after 5 days of treatment. Lumbar puncture 20 days after starting treatments showed decreased white blood cell counts (25 × 10^6^/L). Afterward, the patient received oral methylprednisolone tablets (starting at 48 mg/d, reduced by 8 mg every 2 weeks until 32 mg/d, then decreased by 4 mg every 2 weeks, and ultimately maintained at 12 mg/d) and methylcobalamin tablets (0.5 mg three times a day). About 2 months later, her lower limb muscle strength was normal (grade 5, MRC).

**Table 1:** Laboratory research indicators of the patient

Indicator	Reference	Value
**Cerebrospinal fluid**		
Proteins	0.15–0.45 g/L	0.77 g/L
Red cells	≤0	10 × 10^6^/L
White blood cells	0–15 × 10^6^/L	400 × 10^6^/L
CSF-IgA	0–5 mg/L	12.1 mg/L
CSF-IgG	0–34 mg/L	114 mg/L
CSF-IgM	0-1.3 mg/L	4.39 mg/L
**Plasma**		
LDH	0–30 IU/L	35 IU/L
AQP-4-IgG		Positive 1:320
**Serum immunological examination**		
Antithyroglobulin antibody	0–16 AU/ml	75.5 AU/ml
Thyroperoxidase antibody	0–60 IU/ml	67.2 IU/ml
Anti-SSA-52	0–25 AU/ml	51.71 AU/ml
Anti-mitochondrial antibody-M	0–16 AU/ml	75.5 AU/ml ()
Antinuclear antibody		Positive, 1/1280 cytoplasmic, 1/640 granular.
**Blood cells at 4 months after diagnosis of NOMSD**		
Hemoglobin	110–150 g/L	65 g/L
Platelets	100–300 × 10^9^/L	209 × 10^9^/L
White blood cells	4–10 × 10^9^/L	9.02 × 10^9^/L
Mean corpuscular volume	80–100 fl.	92.1 fl.
Mean corpuscular hemoglobin concentration	320–360 g/L	333 g/L
Reticulocyte	22–84 × 10^9^/L	0.11%

**Fig. 3:**
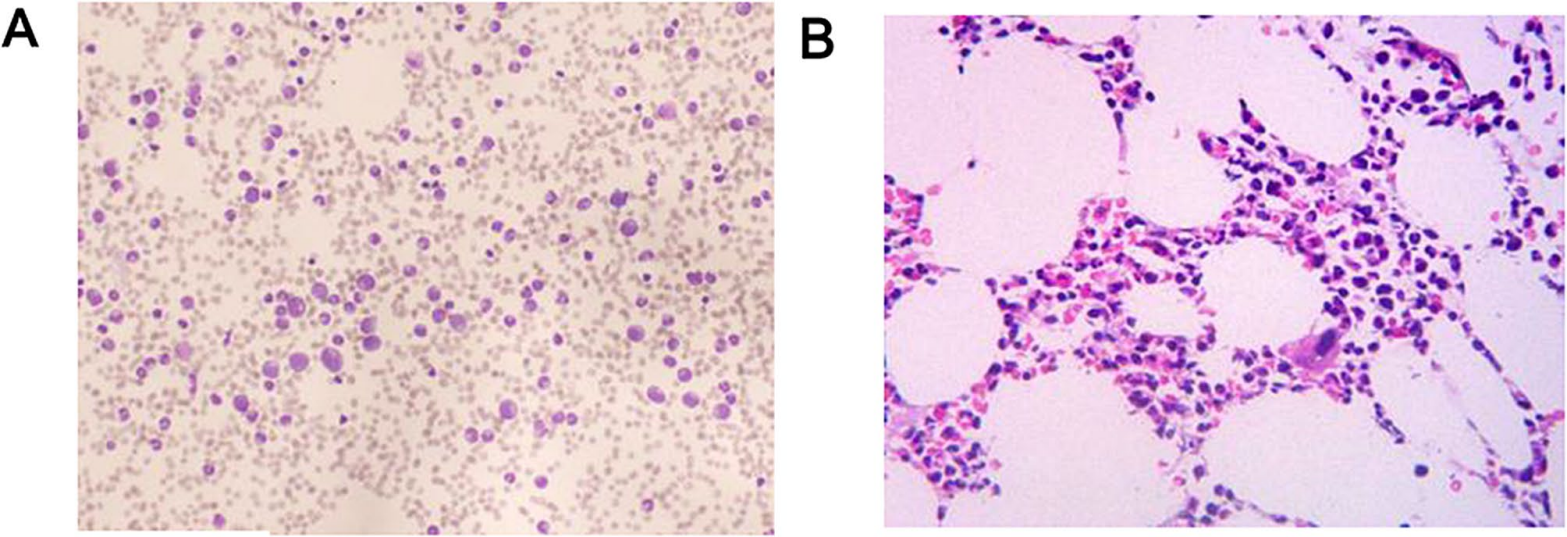
Morphological examination of the bone marrow showed that the patient demonstrated moderate anemia. (**A**) Bone marrow aspirates smear demonstrating predominantly granulocytic precursors with erythrocytoblasts almost invisible (×100, Wright’s staining). (**B**) Bone marrow biopsy showing a normocellular bone marrow, lack of erythroid precursors, granulocytic hyperplasia, and megakaryocytes with a normal appearance (×400, hematoxylin and eosin staining)

In May 2023, the patient was admitted again for fatigue, palpitations, and shortness of breath. Physical examination revealed obvious pallor but no jaundice. Blood cell analysis indicated moderate anemia, increased platelets, and decreased reticulocytes (Table 1). Bone marrow aspiration and biopsy revealed elevated erythroid precursors and erythroid hypoplasia but normal megakaryocytes and myeloid precursors (Fig. 3). PRCA associated with NMOSD was diagnosed. Methylprednisolone (40 mg/day) and cyclosporine (300 mg/day) were given. Recombinant human erythropoietin (EPO) was given (10,000 U once a week for two weeks) to improve anemia symptoms. The patient’s general condition improved after 1.5 months. Hemoglobin and reticulocyte counts were normal (HGB 115 g/L, RET 2.5%). Spinal MRI revealed improvements in the abnormal spinal signals (Fig. 1B).

## Discussion and conclusions

As immune factors can cause changes in hematopoietic function, a comprehensive evaluation of immune function and bone marrow biopsy can be necessary if blood cell abnormalities are observed while managing NMOSD.

NMOSD accompanied by hematological diseases is rare and mostly includes iron-deficiency anemia. Regarding pathogenesis, iron-deficiency anemia may not be directly related to NMOSD but to women of childbearing age [4]. There were also reports of NMOSD with autoimmune hemolytic anemia and immune thrombocytopenia [5, 6]. In the past 20 years, no reports of NMOSD combined with PRCA have been published. In the patient reported here, HGB levels and liver and kidney functions were normal when NMOSD was diagnosed. Three months later, the patient developed moderate anemia (normocytic normochromic), reticulocytopenia, and bone marrow erythrodysplasia but with normal ferritin, folic acid, vitamin B12, and EPO levels and negative DAT test, indicating PRCA.

PRCA is characterized by normocytic normochromic anemia with reticulocytopenia and severely reduced or absence of erythroid precursors in the bone marrow [1]. As this is the first reported case, the pathogenesis of PRCA associated with NOMSD is unknown. It can be speculated that abnormal cellular and humoral immunity leads to changes in the bone marrow microenvironment and abnormal erythroid development. For this patient, not only AQP4-IgG but also ANA, anti-SSA-52, AMA-M2, TRAb, and TPOAb were positive, indicating autoimmune abnormalities. It can be speculated that PRCA and NOMSD are related to autoimmune abnormalities and have a common pathogenesis. Therefore, treatment involved cyclosporine, methylprednisolone, and EPO, leading to HGB recovery.

In conclusion, PRCA can be secondary to NMOSD. A comprehensive immune function and bone marrow biopsy evaluation should be performed if blood cell abnormalities are found while managing NMOSD.

## Data Availability

All data generated or analyzed during this study are included in this article.

## Abbreviations

AMA-M2: Anti-mitochondrial antibody-M2
AQP4: Aquaporin-4
CSF: Cerebrospinal fluid
DWI: Diffusion-weighted imaging
EPO: Erythropoietin
HGB: Hemoglobin
IVIG: Intravenous immunoglobulin
LDH: Lactate dehydrogenase
MRI: Magnetic resonance imaging
NMOSD: Neuromyelitis optica spectrum disorders
PLT: Platelet
PRCA: Pure red cell aplasia
RET: Reticulocyte
STIR: Short-tau inversion recovery
TPOAb: Thyroperoxidase antibody
TRAb: Antithyroglobulin antibody
MRC: Medical Research Council
